# Improving Transparency in the Residency Application Process: Survey Study

**DOI:** 10.2196/45919

**Published:** 2023-12-25

**Authors:** Lindsey Ulin, Simone A Bernstein, Julio C Nunes, Alex Gu, Maya M Hammoud, Jessica A Gold, Kamran M Mirza

**Affiliations:** 1 Department of Medicine Brigham and Women's Hospital Boston, MA United States; 2 Department of Psychiatry Washington University in St Louis St Louis, MO United States; 3 Department of Psychiatry Yale School of Medicine New Haven, CT United States; 4 Department of Orthopaedic Surgery George Washington University School of Medicine Washington, DC United States; 5 Department of Obstetrics and Gynecology University of Michigan Medical School Ann Arbor, MI United States; 6 Department of Pathology and Clinical Laboratories University of Michigan Medicine Ann Arbor, MI United States

**Keywords:** data elaboration, information transparency, medical school, residency application, residency programs, resident

## Abstract

**Background:**

Increasing numbers of residency applications create challenges for applicants and residency programs to assess if they are a good fit during the residency application and match process. Applicants face limited or conflicting information as they assess programs, leading to overapplying. A holistic review of residency applications is considered a gold standard for programs, but the current volumes and associated time constraints leave programs relying on numerical filters, which do not predict success in residency. Applicants could benefit from increased transparency in the residency application process.

**Objective:**

This study aims to determine the information applicants find most beneficial from residency programs when deciding where to apply, by type of medical school education background.

**Methods:**

Match 2023 applicants voluntarily completed an anonymous survey through the Twitter and Instagram social media platforms. We asked the respondents to select 3 top factors from a multiple-choice list of what information they would like from residency programs to help determine if the characteristics of their application align with program values. We examined differences in helpful factors selected by medical school backgrounds using ANOVA.

**Results:**

There were 4649 survey respondents. When responses were analyzed by United States-allopathic (US-MD), doctor of osteopathic medicine (DO), and international medical graduate (IMG) educational backgrounds, respondents chose different factors as most helpful: minimum United States Medical Licensing Examination (USMLE) or Comprehensive Osteopathic Medical Licensing Examination (COMLEX) Step 2 scores (565/3042, 18.57% US-MD; 485/3042, 15.9% DO; and 1992/3042, 65.48% IMG; *P*<.001), resident hometown region (281/1132, 24.82% US-MD; 189/1132, 16.7% DO; and 662/1132, 58.48% IMG; *P*=.02), resident medical school region (476/2179, 22% US-MD; 250/2179, 11.5% DO; and 1453/2179, 66.7% IMG; *P*=.002), and percent of residents or attendings underrepresented in medicine (417/1815, 22.98% US-MD; 158/1815, 8.71% DO; and 1240/1815, 68.32% IMG; *P*<.001).

**Conclusions:**

When applying to residency programs, this study found that the factors that respondents consider most helpful from programs in deciding where to apply differ by educational background. Across all educational groups, respondents want transparency around standardized exam scores, geography, and the racial or ethnic backgrounds of residents and attendings.

## Introduction

An increasing number of residency applications has made it harder for programs and applicants to assess if they are a good fit during the residency application and match process [1]. Residency application volumes continue to increase [1-3] without a change in match outcomes [4]. This increase in applications burdens applicants and residency programs in time spent [5], costs [5-7], and stress [8]. Applicants face limited or conflicting information as they assess programs, leading to overapplying [1,5]. A holistic review of residency applications is considered a gold standard for programs [9,10], but the current volumes and associated time constraints leave programs relying on numerical filters [1,5,9], which do not predict success in residency [11-13]. There are solutions proposed to improve the residency application process, including a cap on the number of applications submitted or interviews accepted [1], preference signaling [14], and a multiphase match process [14-17]. A common theme among these solutions is increasing transparency around program selection criteria [1,2,9]. Current resources for applicants are fraught with biases or missing data and discrepancies [18-21]. This incorrect information is further compounded by mixed messaging from medical school advisors [2,8]. Increased workplace transparency leads to greater job satisfaction [22,23], trust [20], psychological safety [24], decreased intent to leave [23,24], and better commitment and performance [23]. As applying for residency is a process similar to a job application, it should be as transparent and equitable as possible to help both applicants and programs.

Still, increasing transparency is only helpful if we know what information to provide. To our knowledge, no study has identified what information residency applicants would find most helpful in determining where to apply.

**Figure 1:**
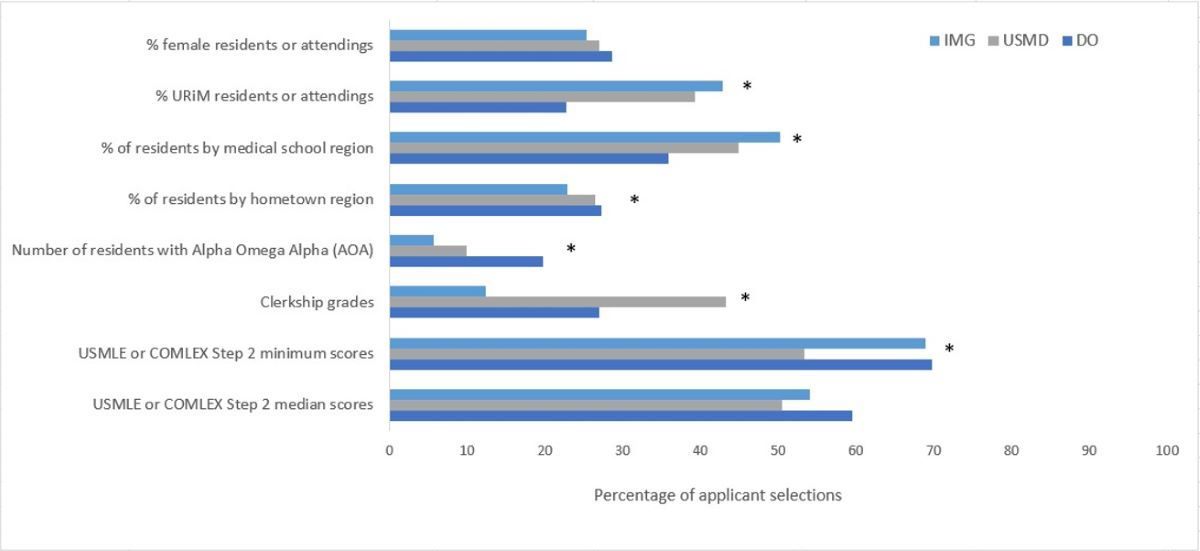
Factors applicants selected as most helpful in assessing program fit and where to apply. * Denotes a difference in the percentage of applicants selecting each factor across the 3 educational backgrounds with a P<.05. COMLEX: Comprehensive Osteopathic Medical Licensing Examination; DO: doctor of osteopathic medicine; IMG: international medical graduate; URiM: underrepresented in medicine; US-MD: United States-allopathic; USMLE: United States Medical Licensing Examination.

## Methods

### Recruitment

Between June 2, 2022, and August 1, 2022, a nonprobabilistic sample of Match 2023 applicants were recruited on the Twitter and Instagram social media platforms. United States-allopathic (US-MD) and doctor of osteopathic medicine (DO) medical school students and international medical graduates (IMGs) applying to US residency programs for Match 2023 were invited to participate. The anonymous open survey was created using Qualtrics (CoreXM version; Qualtrics) and collected demographics including age, gender, race or ethnicity, educational background, and preferred specialty. A link to the survey, announced online on Instagram and Twitter social media posts, directed participants to a Washington University in St Louis Qualtrics website, which did not contain any other information beyond the survey and participant disclosure information.

We asked respondents to select the top 3 factors from a multiple-choice list of what information they would like from residency programs to help determine if the characteristics of their application align with program values. Important factors were used in the survey based on program directors’ reports of factors applicants are evaluated on [25]. An open-ended free response option was included among the listed important factors (Multimedia Appendix 1). A copy of the survey is available in Multimedia Appendix 1; it contains a total of 14 items. Respondents were able to review and change their answers through a Back button. Results from the other questions asked in the survey were analyzed in separate forthcoming work.

### Statistical Analysis

ANOVA was used to assess differences in helpful factors selected by educational background. Free-response factor answers were included in the analysis as the “other” category. SPSS Statistics (version 25; IBM Corp) was used to assess statistical significance (*P*<.05).

### Ethical Considerations

The study was reviewed and deemed exempt by the Washington University in St Louis Institutional Review Board. Privacy and confidentiality were protected through anonymous study data. Participants voluntarily consented to the study, and compensation was not offered. Contact information for the research team and human research protection office were provided.

## Results

The survey was completed by 4649 Match 2023 applicants (Table 1) applying to 25 distinct specialties, with the largest number applying to internal medicine (1502/4649, 32.31%). The survey was started by 5185 respondents, with a completion rate of 89.66% (4649/5185). Incomplete surveys were not included in the data analysis. Overall, 1060/4649 (22.8%) respondents were US-MD, 696/4649 (15%) were DO, and 2893/4649 (62.22%) were IMGs. The overall racial or ethnic distribution identified as Asian (1728/4649, 37.17%), White (970/4649, 20.9%), Hispanic (669/4649, 14.4%), Black (594/4649, 12.8%), American Indian (15/4649, 0.3%), and Pacific Islander (5/4649, 0.1%). When stratified by race or ethnicity, 1283/3981 (32.22%) were underrepresented in medicine (URiM), and 2698/3981 (67.77%) were non-URiM as defined by the Association of American Medical Colleges [26]. Most respondents identified as female (2930/4649, 63.02%) and were aged between 25 and 29 years old (2948/4649, 63.41%).

**Table 1:** Characteristics of applicants, including doctor of osteopathic medicine (DO) students, United States-allopathic (MD) students, and international medical graduates (IMGs).

Characteristic				Total applicants (N=4649), n (%)	DO applicants (n=696), n (%)	US-MD applicants (n=1060), n (%)	IMG applicants (n=2893), n (%)
**Sex or gender**							
	Male			1650 (35.49)	211 (30.3)	359 (33.9)	1080 (37.33)
	Female			2930 (63.02)	473 (68)	675 (63.7)	1782 (61.6)
	Transgender female			4 (0)	2 (0)	1 (0)	1 (0)
	Transgender male			1 (0)	0 (0)	1 (0)	0 (0)
	Gender variant or nonconforming			2 (0)	0 (0)	1 (0)	1 (0)
	Nonbinary			6 (0)	2 (0)	4 (0)	0 (0.0)
	Prefer not to answer			56 (1)	8 (1)	19 (1)	29 (1)
**Race or ethnicity**							
	American Indian			15 (0)	1 (0)	1 (0)	13 (1)
	Asian			1728 (37.17)	176 (25.3)	261 (24.6)	1291 (44.62)
	Black, African American, or African			594 (12.8)	39 (5)	174 (16.4)	381 (13.2)
	Hispanic or Latino			669 (14.4)	62 (8)	162 (15.3)	445 (15.4)
	Native Hawaiian or other Pacific Islander			5 (0)	0 (0)	1 (0)	4 (0)
	White			970 (20.9)	343 (49.3)	353 (33.3)	274 (9.5)
	Multiple			119 (2.6)	24 (3)	40 (3)	55 (2)
	Other			328 (7.1)	25 (3)	27 (2)	276 (9.5)
	Prefer not to answer			221 (4.8)	26 (3)	41 (4)	154 (5.3)
URiM^a^				1283 (32.22)	102 (16.4)	338 (35.5)	843 (35)
Non-URiM^a^				2698 (67.77)	519 (83.6)	614 (64.5)	1565 (65)
**Age (years)**							
	19-24			648 (13.9)	70 (10)	131 (12.4)	447 (15.5)
	25-29			2948 (63.41)	527 (75.7)	796 (75.1)	1625 (56.17)
	30-34			783 (16.8)	84 (12)	116 (10.9)	583 (20.2)
	35-39			192 (4.1)	11 (2)	14 (1)	167 (5.8)
	≥40			78 (2)	4 (0)	3 (0)	71 (23)

^a^URiM: underrepresented in medicine; In accordance with the Association of American Medical College definition of URiM. URiM is defined as racial and ethnic populations that are underrepresented in the medical profession relative to the general population. This definition is expanded from the 2003 term that previously specified Black, Mexican-American, Native American (American Indian, Alaska Native, or Native Hawaiian), and mainland Puerto Ricans [26]. Applicants who identified as “multiple races or ethnicities” and “preferred not to answer” were removed to calculate the number of URiM and non-URiM respondents.

When looking at the top 3 factors that would be most helpful to decide where to apply, there were differences in the percentages of respondents selecting “minimum United States Medical Licensing Examination (USMLE) or Comprehensive Osteopathic Medical Licensing Examination (COMLEX) Step 2 scores” (563/3042, 18.57% US-MD, 485/3042, 15.9% DO, and 1992/3042, 65.48% IMG; *P*<.001), “resident hometown region” (281/1132, 24.82% US-MD, 189/1132, 16.7% DO, and 662/1132, 58.48% IMG; *P*=.02), and “percent of URiM residents or attendings” (417/1815, 22.98% US-MD, 158/1815, 8.71% DO, and 1240/1815, 68.32% IMG; *P*<.001) between respondents from the 3 educational backgrounds. Table 2 shows differences in all factors. A higher percentage of URiM respondents across all educational backgrounds consistently selected the “percentage of URiM residents and attendings” as factors that would be most helpful to decide where to apply, compared to non-URiM respondents (903/1283, 70.38% URiM vs 655/2698, 24.28% non-URiM; *P*<.001) ranked it as an important factor.

**Table 2:** Factors applicants (N=4649) selected as most helpful in assessing program fit and where to apply.

Factors	IMG^a^ applicants (n=2893), n (%)	US-MD^b^ applicants (n=1060), n (%)	DO^c^ applicants (n=696), n (%)	*P* value
Percentage female residents or attendings	735 (25.4)	286 (27)	199 (28.6)	.32
Percentage URiM^d^ residents or attendings	1240 (42.9)	417 (39.3)	158 (22.7)	<.001
Percentage of residents by medical school region	1453 (50.2)	476 (44.9)	250 (35.9)	.002
Percentage of residents by hometown region	662 (22.9)	281 (26.5)	189 (27.2)	.02
Number of residents in Alpha Omega Alpha	163 (5.6)	105 (9.9)	138 (19.8)	<.001
Clerkship grades	357 (12.3)	458 (43.2)	188 (27)	<.001
USMLE^e^ or COMLEX^f^ Step 2 minimum scores	1992 (68.9)	565 (53.3)	485 (69.7)	<.001
USMLE or COMLEX Step 2 median scores	1562 (54.0)	535 (50.5)	414 (59.5)	0.49

^a^IMG: international medical graduate.

^b^US-MD: United States-allopathic.

^c^DO: doctor of osteopathic medicine.

^d^URiM: underrepresented in medicine.

^e^USMLE: United States Medical Licensing Examination.

^f^COMLEX: Comprehensive Osteopathic Medical Licensing Examination.

## Discussion

### Principal Results

The factors that respondents find most helpful in deciding which residency programs to apply to differ by educational background (US-MD, DO, and IMG), but the most highly sought are around USMLE or COMLEX Step 2 scores, hometown regions of residents, and the racial or ethnic background of residents and attendings. Some of this information is available on databases like the Fellowship and Residency Electronic Interactive Database (FREIDA) [27], the Orthopaedic Residency Information Network (ORIN) [28], and program websites, but some of it is outdated [18-21]. Given that some programs screen applications by test scores [1,5,9], it would be helpful for applicants to have updated information about a program’s review criteria. Geographic preferences may also play a large role in the match process [29]. For the 2021 Match, there was a significant increase in matched applicants to their home orthopedic surgery and integrated plastic surgery programs [30,31]. Additionally, programs likely consider that more than half of residents practice where they train [32]. Consequently, increased transparency about geographic interests as an optional addition to the application could benefit programs to improve resident retention over time and for applicants to show interest in specific areas of the country. In the Match 2023, a total of 16 specialties asked about geographic preferences in the supplemental application, and program directors expressed positive feedback on its inclusion [33,34]. As of the Match 2024, the optional geographic interests section has been expanded to all specialties, representing an initial step in allowing applicants to express interest in those programs who consider geography important in their decision-making process.

Structural racism persists in medical training and match processes [35]. Residents and attendings who identify as URiM routinely experience bias at work and are relied on to fulfill a lack of standardized systems to promote diversity [36]. Although a higher percentage of URiM respondents selected the percentage of URiM faculty and residents as important in assessing fit, many non-URiM applicants selected it, too. Both groups likely value a safe and inclusive work environment for themselves or their colleagues. It is important to recognize various intersectional aspects of diversity, equity, and inclusion in the match to mitigate perceived (and real) risk for applicants; however, there is little best practice data for programs to counteract inequities of the application process. Some strides toward greater equity and inclusivity for the match include the addition of pronouns to the Electronic Residency Application Service. In addition, some programs may choose to screen out identifiers such as date of birth, headshots, and hometown when reviewing applications [37]. While additional data can benefit applicants, it can also help advisors provide tailored information. Metrics such as test scores, clinical grades, and experiences from previous applicants can be used to guide applicants on programs to apply to. This method of advising can help applicants strategically apply where they are likely to match, which may decrease anxiety, expense, and overapplying [38]. Growing internal and external databases like FREIDA [27] or the Texas Seeking Transparency in Application to Residency (STAR) [39], which contain aggregated data from recently matched medical students, can provide additional guidance. Expanding access to the Texas STAR by removing the requirement that medical schools must share their data to gain access and increasing IMG-affiliated medical schools’ participation, may increase transparency and improve match outcomes. Expanding specialty-specific databases to all specialties would be helpful, like ORIN [28] for orthopedic surgery and the Alignment Check Index [40] for obstetrics and gynecology. Using information from this study, updating these databases with the information applicants are most interested in may provide a better assessment of applicant fit and decrease overapplying. In addition to the percentage of URiM residents and attendings, which applicants selected as a helpful factor, programs can update their website and centralized databases with greater diversity, equity, and inclusion content [41], including information about URiM away rotation scholarship opportunities [42].

### Limitations

Limitations of this study include self-selection bias through the use of social media to publicize the survey. However, the study has a large applicant pool; we detected differences among respondents based on educational backgrounds.

### Conclusions

There have been numerous calls to improve the residency application and match process as application volumes increase. A common theme in the solutions proposed is improving transparency and equity in the process, but only some have highlighted how. Although we found differences across educational backgrounds in the top factors that respondents would find most helpful from programs in deciding where to apply, many want information on the hometown region of residents, test scores, and the diversity of programs, which programs can consider adding to centralized residency databases. Increasing transparency may help applicants determine the programs that align best with their application and interests and improve the guidance from medical school advisors.

## Appendix

Multimedia Appendix 1 The anonymous survey completed by residency applicants querying helpful factors for assessing programs for the 2023 Match.

## Abbreviations

COMLEX: Comprehensive Osteopathic Medical Licensing Examination
DO: doctor of osteopathic medicine
FREIDA: Fellowship and Residency Electronic Interactive Database
IMG: international medical graduate
ORIN: Orthopaedic Residency Information Network
STAR: Seeking Transparency in Application to Residency
URiM: underrepresented in medicine
US-MD: United States-allopathic
USMLE: United States Medical Licensing Examination

## Data Availability

Data used in this analysis are available from the corresponding author upon reasonable request.
