# Supplementary material for: Improving Transparency in the Residency Application Process: Survey Study
Source: JMIR Form Res. 2023 Dec 25;7:e45919. doi: 10.2196/45919 (PMC10775039; doi:10.2196/45919)
Supplement: Multimedia Appendix 1 [file formative_v7i1e45919_app1.docx]

Survey

Start of Block: Default Question Block

We invite you to participate in a research study being conducted by investigators from Washington University in St. Louis. If you have questions for the research team, please contact Simone A. Bernstein, MD simone.bernstein@wustl.edu, or you may contact the Human Research Protection Office at 1-(800)-438-0445 or hrpo@wustl.edu. Thank you very much for your consideration of this research study.

Where do/did you attend medical school?

- United States Osteopathic Medical School (DO)
- United States Allopathic Medical School (MD)
- International Medical Graduate

What specialty do you plan to apply to in the match? Select your preferred specialty.

- Anesthesiology
- Cardiothoracic Surgery
- Dermatology
- Diagnostic Radiology
- Emergency Medicine
- Family Medicine
- General Surgery
- Internal Medicine
- Med-Peds
- Neurology
- Neurosurgery
- Obstetrics & Gynecology
- Ophthalmology
- Orthopedic Surgery
- Otolaryngology
- Pathology
- Pediatrics
- Physical Medicine & Rehabilitation
- Plastic Surgery
- Preventive Medicine
- Psychiatry
- Radiation Oncology
- Urology
- Vascular Surgery
- Other __________________________________________________

Age.

- 19-24
- 25-29
- 30-34
- 35-39
- ≥40

| Page Break |  |
| --- | --- |

Race/ethnicity.

- American Indian
- Asian
- Black, African American, or African
- Hispanic, Latino
- Native Hawaiian or other Pacific Islander
- White Caucasian
- Multiple races/ethnicities
- Other __________________________________________________
- Prefer not to answer

Gender identity.

- Male
- Female
- Transgender female
- Transgender male
- Gender variant/non-conforming
- Non-binary
- Prefer not to answer

| Page Break |  |
| --- | --- |

Personal background. (select all that apply)

- First in family to graduate college
- First in family to attend graduate school
- First-generation immigrant
- Recipient of government benefits (Medicaid or SNAP recipients)
- English was not my first language
- Identify as having a disability
- Someone in my immediate family is a physician
- ⊗None apply to me

Do you perceive any obstacles based on your racial identity, gender identity, or personal background for the upcoming match application process in comparison to others?

- Yes
- No

Display This Question:

If Do you perceive any obstacles based on your racial identity, gender identity, or personal backgro... = Yes

Please describe how obstacles based on your racial identity, gender identity, or personal background will likely affect the match application process.

________________________________________________________________

How satisfied are you with the quality and amount of guidance provided about the residency application process <b>by your medical school</b>?

- Very Unsatisfied
- Unsatisfied
- Neutral
- Satisfied
- Very Satisfied

| 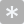 |
| --- |

Where do you receive most of your guidance about how to apply to residency? Select the <b>top three resources</b> where you receive the most information.

- Inside The Match
- Association of American Medical Colleges (AAMC) Website
- Reddit
- Medical school deans
- Medical school peers
- Academic advisor
- Faculty mentor
- Resident physicians
- Family
- Other (please specify) __________________________________________________

| 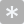 |
| --- |

What resources will you use to select which programs to apply to for residency? Select the <b>top three resources</b> you plan to receive the most information about residency programs.

- Residency program websites
- Residency program social media pages
- Virtual Open Houses/Residency Fairs
- Doximity Rankings
- 2021 NRMP Program Director Survey
- Reddit
- FRIEDA (Fellowship and Residency Electronic Interactive Database)
- Residency Explorer
- Faculty mentor
- Alumni from my medical school
- Resident physicians in programs I want to apply to
- Other (please specify) __________________________________________________

| 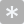 |
| --- |

If residency programs provide more information for you to better decide if you were a good fit for that program, what information would be most helpful for you to make decisions about where to apply? <b>Select your top three choices.</b>

- Minimum USMLE/COMLEX Step 2 scores
- Median USMLE/COMLEX Step 2 scores of residents
- Third-year and fourth-year clerkship grades
- Number of residents with AOA
- Percentage of residents by hometown region
- Percentage of residents by medical school region
- Percentage of female residents and attendings
- Percentage of unrepresented in medicine residents and attendings
- Other (please specify) __________________________________________________

How satisfied are you with the way your <b>medical school emotionally prepares</b> you for the <b>possibility of being unmatched</b>?

- Very Unsatisfied
- Unsatisfied
- Neutral
- Satisfied
- Very Satisfied

How satisfied are you with the way your medical school prepares you <b>informationally and logistically </b>for the<b> possibility of being unmatched</b>?

- Very Unsatisfied
- Unsatisfied
- Neutral
- Satisfied
- Very Satisfied

Is there anything else that would help you prepare for the residency application process?

________________________________________________________________
